# Time to Loss of Consciousness and Its Relation to Behavior in Slaughter Pigs during Stunning with 80 or 95% Carbon Dioxide

**DOI:** 10.3389/fvets.2016.00038

**Published:** 2016-05-19

**Authors:** Merel Verhoeven, Marien Gerritzen, Antonio Velarde, Ludo Hellebrekers, Bas Kemp

**Affiliations:** ^1^Animal Welfare Department, Animal Sciences Group, Wageningen University and Research Centre, Wageningen, Netherlands; ^2^Adaptation Physiology Group, Animal Sciences Group, Wageningen University, Wageningen, Netherlands; ^3^Animal Welfare Subprogram, IRTA, Girona, Spain; ^4^Central Veterinary Institute, Animal Sciences Group, Wageningen University and Research Centre, Wageningen, Netherlands

**Keywords:** animal welfare, behavior, carbon dioxide, electroencephalogram, pigs, stunning

## Abstract

Exposure to CO_2_ at high concentration is a much debated stunning method in pigs. Pigs respond aversively to high concentrations of CO_2_, and there is uncertainty about what behaviors occur before and after loss of consciousness. The aim was to assess timing of unconsciousness in pigs during exposure to high concentrations of CO_2_ based on changes in electroencephalogram (EEG) activity and the relation with the behaviors sniffing, retreat and escape attempts, lateral head movements, jumping, muscular contractions, loss of posture, and gasping. Pigs (108 ± 9 kg) were randomly assigned to 80% CO_2_ (80C, *n* = 24) or 95% CO_2_ (95C, *n* = 24). The time at which the gondola started descending into the well pre-filled with 80C or 95C was marked as *T* = 0. The CO_2_ exposure lasted 346 s after which the corneal reflex and breathing were assessed for 1 min. Visual assessment of changes in the amplitude and frequency of EEG traces after *T* = 0 was used to determine loss of consciousness. Time to loss of consciousness was longer in 80C pigs (47 ± 6 s) than in 95C pigs (33 ± 7 s). Time to an iso-electric EEG was similar in 80C pigs (75 ± 23 s) and 95C pigs (64 ± 32 s). When pigs descended into the well, the earlier entry of 95C pigs into high CO_2_ atmosphere rather than the concentration of CO_2_ by itself affected the latency of behavioral responses and decreasing brain activity. During exposure to the gas, 80C and 95C pigs exhibited sniffing, retreat attempts, lateral head movements, jumping, and gasping before loss of consciousness. 95C pigs exhibited all these behaviors on average earlier than 80C pigs after *T* = 0. But the interval between onset of these behaviors and loss of consciousness and the duration of these behaviors, except gasping, was similar for both treatments. Loss of posture was on average observed in both groups 10 s before EEG-based loss of consciousness. Furthermore, 88% of 80C pigs and 94% of 95C pigs demonstrated muscular contractions before loss of consciousness. The findings provide little reason to conclude on a behavioral basis that these atmospheres are greatly different in their impact on pig welfare.

## Introduction

The two most commonly used stunning methods applied under commercial slaughterhouse conditions in pigs are electrical stunning and exposure to high concentrations of carbon dioxide (CO_2_) (1). The CO_2_ stunning method involves lowering groups of pigs in a gondola into a well that is pre-filled with a high concentration of CO_2_. According to European legislation, the CO_2_ concentration should at least be 80%, but many slaughterhouses use 90% CO_2_ or higher in attempts to increase throughput at the slaughter plant (2, 3). Rapid and deeper respiration induced by higher CO_2_ concentrations increases the intake of CO_2_ that shortens the induction period and time to loss of consciousness (4). Induction of unconsciousness with CO_2_ stunning requires high concentrations of CO_2_ where excessive CO_2_ concentrations in the blood lead to a state of hypercapnic hypoxia, inducing a decline in blood pH levels. Because CO_2_ travels across the blood–brain barrier relatively easy, the high CO_2_ levels also cause rapid acidification of the cerebrospinal fluid. The drop in pH is detected by central chemical receptors in the medulla oblongata and pons of the brainstem, resulting in faster and deeper respiration in an attempt to increase pO_2_ and decrease pCO_2_ (5). The acidification of the brain cells results in a depression of brain activity that causes loss of consciousness or when prolonged death (6). Loss of consciousness is not immediate upon exposure to high CO_2_ levels, but depends on the CO_2_ concentration used and the speed at which animals are immersed into the highest concentration of CO_2_ at bottom of the well (7, 8). Time to loss of posture, as the first indicator of the onset of unconsciousness, was reported at 25, 17, 22, and 15 s after immersion into 60, 70, 80, and 90% CO_2_, respectively (7). Studies that examine brain activity, presented in an electroencephalogram (EEG), reported loss of consciousness 14–60 s after initial exposure to 80–90% CO_2_ (9, 10). Pigs do not need to be individually restrained and can be stunned in groups during CO_2_ stunning, which are considered to be advantages in terms of animal welfare in comparison to other stunning methods (10, 11). Before pigs lose consciousness, however, behavior, including excitement, retreat and escape attempts, and respiratory changes (gasping), has been observed (3, 7, 12, 13). Carbon dioxide itself causes irritation of nasal mucosal membranes and is a strong respiratory stimulator that induces a sense of breathlessness prior to loss of consciousness in humans (14, 15). Beausoleil and Mellor (16) describe three different traits of breathlessness, namely chest tightness, respiratory effort, and air hunger, where air hunger occurs when the demand for ventilation exceeds the capacity to provide it (17). This air hunger may occur when an animal continuously inhales high tensions of CO_2_ and is a serious concern for animal welfare as it always unpleasant to the animal (16).

Furthermore, there is much debate about what CO_2_ concentrations are most aversive to pigs since behavioral responses of pigs vary with different CO_2_ concentrations. Pigs seem to respond less aversive to lower concentrations of CO_2_ (50–60%) than higher concentrations of CO_2_ (80–90%) (7, 18, 19). When looking at these high concentrations of CO_2_, Nowak et al. (20) observed higher lactate levels, indicative of stress, in pigs exposed to 80% CO_2_ compared to pigs exposed to 90% CO_2_. Barfod (21) and Erhardt et al. (22), on the other hand, did not find conclusive evidence that CO_2_ irritates the membranes and concluded that gasping is a normal response to the excessive CO_2_ in the body. The general opinion, however, is that the initial phase of CO_2_ stunning is aversive to pigs (20).

While changes in breathing pattern are generally associated with aversion, there is little consensus concerning the interpretation of the occurrence of convulsions or (involuntary) muscle contractions (13). These muscle contractions have been observed both before (3, 9) and after loss of consciousness (3, 23, 24). The objective of the current study was to assess the relationship between behavioral measurements and onset of unconsciousness as identified by EEG activity during 80% CO_2_ (80C) or 95% CO_2_ (95C) stunning in pigs.

## Materials and Methods

This study was approved by the Animal welfare body of Wageningen UR, The Netherlands and by the Institutional Animal Care and Use Committee (IACUC) of IRTA, Spain.

### Animals and Housing

In total, 48 cross breed (Pietrain × Large White × Landrace) pigs from a commercial fattening farm were randomly selected and transported to the experimental facilities. Before departure from the farm, all animals were systematically inspected by clinical examination of physical appearance and the normality of behavior, removing those presenting signs of disease. The selected pigs (live weight 108 ± 9 kg) arrived at the experimental facilities 3 days prior to start of the experiment and were housed in groups of eight in six adjacent lairage pens of 4.5 m × 1.8 m, next to the experimental abattoir. The pigs had free access to water and were fed (3 kg/pig/day) twice daily at 0700 and 1600 h using the same commercial diet they received on the fattening farm.

### Experimental Set-up

From the day of arrival until the beginning of the experiment, pigs were habituated to human contact twice a day for 5 min per pen. The experiment was conducted on four consecutive days, starting 3 days after arrival of the pigs. The CO_2_ stunning unit was a dip-lift system (Butina Aps, Copenhagen, Denmark) that contained a gondola (299 cm × 138 cm × 100 cm) that descended to the base of a well at a depth of 290 cm. On the first 2 days, pairs of pigs (always the same pairs randomly selected from the same lairage pen) were habituated to the ascending and descending of the dip-lift (once every day) containing atmospheric air. All pigs were equipped with EEG electrodes and a respiratory band each day, before they entered the gondola. In order to confirm that there was no effect of being in a (ascending or descending) gondola on EEG activity, data were recorded in 24 of the 48 pigs (one pig from each pair). The descent of the gondola took 23 s, where it remained at the bottom of the well for 30 s before ascending in 23 s. The total cycle lasted 76 s and when the gondola reached the top, the pigs were allowed to exit the gondola and the recording equipment was removed. Thereafter, pigs were allowed to return to their pen. On the third or fourth day, the same pairs of pigs, equipped with EEG electrodes and a respiratory band, were again placed in the dip-lift gondola and exposed to the stunning treatments. The well was pre-filled with CO_2_ through an inlet valve at the bottom of the well and the CO_2_ concentration was pre-set and measured using a sensor placed at a depth of 2.5 m. After the complete experiment had finished, CO_2_ concentrations were measured once at five different depths into the well while the well was pre-filled with 80C or 95C. The gondola contained 80C on the first morning and second afternoon, and 95C on the first afternoon and second morning of days 3 and 4. Descent of the gondola took 23 s, before remaining stationary at the bottom for 300 s before ascending in 23 s. The total cycle lasted 346 s and when the gondola reached the top, the exit gate was opened and pigs were assessed for signs of return to consciousness. The corneal reflex was assessed at 10 s intervals and occurrence of breathing was assessed continuously for 60 s. Thereafter, the EEG electrodes and the respiratory band were removed and each pig was bled and sent for further processing.

### Electroencephalogram Activity and Respiratory Signal Measuring Procedure

**Figure 1:**
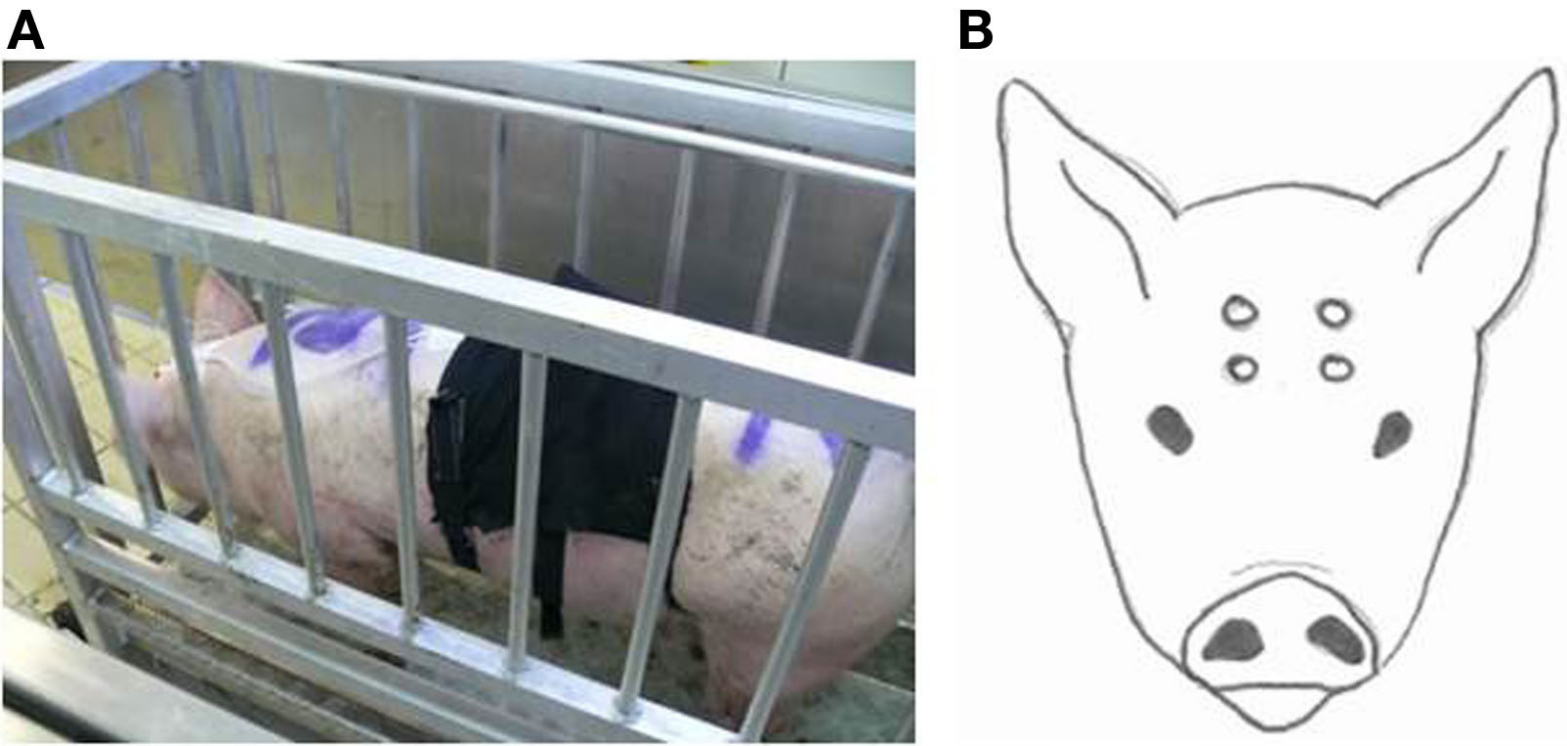
**Weighing scale in which the pigs were fixated (A) to equip them with EEG-recording equipment and a respiratory band**. Placement of the four electrodes on the pigs head **(B)**.

To facilitate instrumentation, pigs were fixated in a standing position in a weighing scale (Figure 1A). A nose clamp or any other additional restraining method was not required. During instrumentation, pigs remained in the weighing scale for approximately 10 min. The head of the animal was shaved on day 1 with an electrical trimmer to enable placement of EEG electrodes. Four Ag/Cl electrodes [Twente Medical Systems International (TMSi), Oldenzaal, The Netherlands] were placed on the shaved skin after applying adhesive tissue (3M Vetbond™, St. Paul, ME, USA). Two electrodes were placed on the skin of the forehead, 2 cm left and right from the sagittal midline and 2 cm below a line extending between the base of both ears. The other two electrodes were placed on the frontal bone 2 cm left and right from the sagittal midline 3 cm frontal from the first electrodes (Figure 1B). All electrodes were connected via a 140 cm active protected cable to a 6-channel Mobi system (TMSi, Oldenzaal, The Netherlands). The Mobi system uses bipolar amplifier technology with high input impedance (>1 GΩ) that amplifies the potential difference between each pair of electrodes. The input amplifier is dimensioned as multichannel instrumentation amplifier. Electrode impedance was <5 kΩ. The EEG was displayed with a band pass filter of 0.5 and 30 Hz, respectively, and unfiltered data were saved onto a computer. Sampling rate was set at 1 kHz. Once the electrodes had been secured and a good live signal was obtained in the weighing scale, baseline EEG activity was recorded for at least 2 min. The gondola started descending into the well at *T* = 0. Recording of the EEG was continuous until the pig left the gondola (days 1 and 2) or was bled (day 3 or 4).

A respiratory waveform was recorded continuously by placing an inductive respiratory band (80 cm) around the abdomen behind the pig’s last rib (TMSi, Oldenzaal, The Netherlands).

### Behavioral Measurements

Pig behavior in the gondola was recorded using two video cameras (Sony Colour CD AVC 565, Circontrol, Barcelona, Spain) placed on the top of the gondola and were connected to a digital image recorder (VDVR-4S 550430, Circontrol).

After at least 2 min of baseline recording in the weighing scale, pigs were gently moved to the gondola. After the gondola, containing two pigs, descended into the well (*T* = 0), number (events), duration (states), and latency to the behaviors as defined in Table 1 were scored per pig from the video recordings using Observer 5.0 software (Noldus Information technology B.V., Wageningen, The Netherlands).

**Table 1:** **Ethogram used to score the behaviors of pigs in an ascending and descending gondola into a well filled with atmospheric air on days 1 and 2 and 80 or 95% carbon dioxide (CO_2_) on days 3 and 4**.

Behavior (event)	Description
Sniffing	Sniffing while lifting the head and considered a first sign of the pig becoming aware of the CO_2_
Retreat attempts	Pigs backing away (18)
Gasping	A very deep breath through a wide open mouth that may involve stretching of the neck (25)
Escape attempts	Pigs running across the gondola and/or raising their forelegs on the side wall of the gondola (7)
Jumping	Jumping in air or against the wall of the gondola
Lateral head movements	Head movements to the side while convulsive expulsion of air from the lungs through the nose and mouth (26)
Muscular contractions	Defined as a period of struggling ranging from fairly vigorous running and movements to clonic convulsive seizures (18)
Loss of posture	The pig is in a recumbent position with total loss of control of posture

**Behavior (state)**	**Description**

Standing	The pig is in an upright position, without moving, with all four paws on the floor
Walking	The pig moves in a forward direction
Sitting	The pig is in a sitting position
Lying	The pig is in a recumbent position, still having (partially) control of posture (it may lift the head)

### Data Analyses

**Figure 2:**
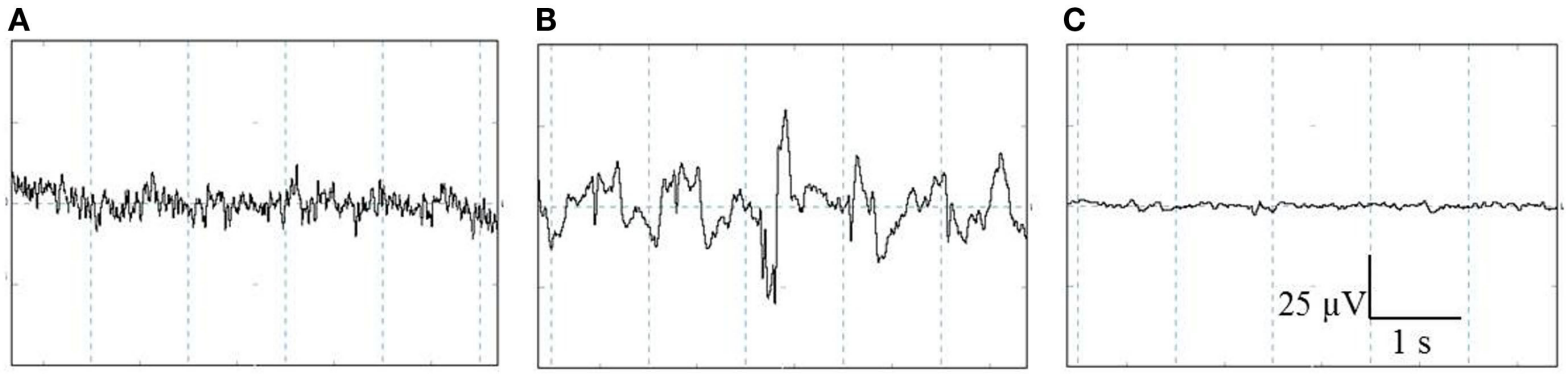
**Representative examples of the different stages identified with visual assessment of electroencephalogram (EEG) activity prior to and after CO_2_ stunning in pigs**. The three stages from left to right: baseline **(A)**, unconscious **(B)**, and minimal brain activity **(C)**. Total *X*-axis represents 5 s, *Y*-axis represents amplitude of the EEG-trace (microvolts).

All EEG data were displayed, stored, and analyzed using PolyBench software (TMSi, Oldenzaal, The Netherlands). The EEG activity (amplitude and frequency) of each pig was visually assessed to determine robust changes in the individual stages, i.e., baseline, unconscious, and minimal brain activity. The baseline stage consisted of a low-amplitude, high-frequency signal, indicating alert pigs (Figure 2A) (27, 28). When high amplitude, low frequency dominated the EEG trace, this was interpreted to indicate unconsciousness (Figure 2B) (27–29). Minimal brain activity was reflected by a flat signal (<10% of baseline amplitude) (Figure 2C) (28). Latency to first apnea was defined as the first time the respiratory waveform signal was flat for at least 5 s. Respiratory arrest was defined as the time at which the respiratory waveform signal stayed flat until the end of the experiment.

### Statistical Analyses

Behaviors and EEG variables were analyzed in SAS 9.3 (SAS Inst. Inc. Cary, NC, USA). To assess whether behavior differed between the 2 days during which the gondola only contained atmospheric air, the following behaviors: walking, standing, and lying expressed as percentages were analyzed with either PROC MIXED when normality was approximated (standing, lying) or PROC GLIMMIX when normality could not be approximated (walking). In PROC GLIMMIX, a binomial distributions with the default logit link was used. As behaviors were assessed on 2 days for each animal, observations cannot be considered independent. Therefore, a repeated measurement analysis was performed with animals as the subject applying a first-order auto-regressive [AR(1)] variance–covariance structure determined to be the best fit according Akaike’s corrected information criterion. The model included the fixed class effect of day (days 1 and 2) and the random effect of the experimental unit pair (pair 1–24) nested within days (model 1).

To assess effects of CO_2_ treatment and possible day effects on latencies to, and durations of, behaviors, standing, walking, sitting, lying, sniffing, retreat and escape attempts, gasping, lateral head movements, jumping, loss of posture, and muscular contractions were analyzed using PROC MIXED. The fixed class effect of treatment (80C, 95C), day (days 3 and 4), and their interaction were assessed. A random effect of the experimental unit pair nested within day and treatment was added to the model (model 2). For retreat attempts, lateral head movements, and jumping, normality was not attained and the natural logarithm of the respective variables was calculated to approximate normality.

To assess effects of CO_2_ treatment and possible day effects on the prevalence of behaviors, i.e., retreat attempts, gasping, lateral head movements, jumping and muscular contractions, assessed as a binary trait, were analyzed with PROC GLIMMIX with a binary distribution and a logit link. The model included the same fixed and random effects specified in model 2. Results are displayed as mean ± SD, unless stated otherwise. In all cases, significance was assumed at *P* < 0.05.

## Results

### Air Treatment

#### Electroencephalogram

Baseline EEG activity was representative for conscious and awake animals in all pigs (*n* = 24): high-frequency, low-amplitude waves as depicted in Figure 2A. No differences in EEG stadia were observed during the baseline period or descending and ascending of the gondola for pigs in the first 2 days.

#### Behavior

The percentage of time spent on walking was similar (*P* > 0.10) for pigs on day 1 (11 ± 9%) compared to day 2 (9 ± 7%) during the 76 s in the gondola. The percentage of time spent on standing was also similar (*P* > 0.10) for pigs on day 1 (89 ± 9%) compared to day 2 (91 ± 7%) during the 76 s in the gondola. None of the pigs were observed sitting or lying and no gasping, jumping, muscular contractions, or escape attempts were observed on these 2 days.

### CO_2_ Treatment

#### CO_2_ Concentration

The average CO_2_ concentration at a depth of 2.5 m was lower (*P* < 0.0001) during the 12 runs of 80C (82 ± 2.1%) than during the 12 runs of 95C (97 ± 0.5%).

Table 2 shows CO_2_ concentrations at five different depth levels in the well for the 80C and 95C treatment measured once after the experiment had taken place.

**Table 2:** **CO_2_ concentrations measured at different levels during descend of the gondola during the two treatments (80C and 95C)**.

Level of the sensor^a^	Treatment^c^		
	Time (s)^b^	80C (%)	95C (%)
0 m (top)	0	1.5	24.5
0.5 m	4	2.7	74.0
1 m	8	7.5	88.2
2 m	16	70.2	96.0
2.5 m	23	79.4	96.9

*^a^Placement of the CO_2_ sensors measured from the top where the animals entered the gondola (0 m)*.

*^b^Time taken from the top where the animals entered the gondola. *T* = 0 is start descending the gondola*.

*^c^Pigs were exposed to either 80% CO_2_ (80C) or 95% CO_2_ (95C) measured at the bottom of the well*.

#### Electroencephalogram

One 95C pig was not equipped with EEG electrodes since this pig was too restless during application of the equipment. Baseline EEG activity was successfully recorded in all other pigs and was representative for conscious and awake animals: high-frequency, low-amplitude waves (Figure 2A). During the CO_2_ exposure phase, in nine animals, the EEG signal was lost due to muscular contractions during the stunning procedure. Of the 38 remaining continuously recorded animals, 20 were exposed to 80C and 18–95C. In one pig exposed to 80C and two pigs exposed to 95C, time to unconsciousness could not, but time to an iso-electric EEG could, be determined due to muscular contractions. In two pigs exposed to 80C, time to unconsciousness could, but time to an iso-electric EEG could not be determined due to muscular contractions.

Time to loss of consciousness based on EEG activity was longer (*P* < 0.001) in 80C pigs (47 ± 6 s; range 39–61 s) than in 95C pigs (33 ± 7 s; range 21–44 s). Time to an iso-electric EEG did not differ (*P* = 0.39) between 80C pigs (75 ± 23 s; range 54–150 s) and 95C pigs (64 ± 32 s; range 36–132 s).

#### Respiration

A good respiratory waveform signal lasting the entire CO_2_ treatment was successfully recorded in 31 pigs. In the other seven pigs, the respiratory waveform could not be detected due to muscular contractions. Of the successfully recorded pigs, 17 were exposed to 80C and 14 were exposed to 95C. Time to the first apnea was longer (*P* < 0.001) in 80C pigs (71 ± 14 s; range 52–103 s) than in 95C pigs (44 ± 7 s; range 33–56 s). Time to respiratory arrest was longer (*P* = 0.001) in 80C pigs (235 ± 61 s; range 151–337 s) than in 95C pigs (152 ± 39 s; range 96–209 s).

#### Behavior

Table 3 shows the latency to first, duration of (mean ± SD), and number of behaviors observed in pigs exposed to 80C and 95C for 346 s. No difference in walking was observed between 80C and 95C pigs. Both sitting and lying occurred earlier (*P* < 0.001) in 95C pigs compared to 80C pigs and duration of standing was shorter (*P* < 0.001) in 95C pigs compared to 80C pigs. Latency to all event behaviors (sniffing, gasping, retreat attempt, lying, muscular contractions, and loss of posture) was longer (*P* < 0.0001) in 80C pigs than 95C pigs.

**Table 3:** **Latency to first, duration of (mean ± SD), and number of behaviors observed in pigs exposed to 80% CO_2_ and 95% CO_2_ for 346 s**.

Behavior^1^	80% CO_2_		95% CO_2_	
	*N*^2^	Mean ± SD	*N*	Mean ± SD
**States**				
Duration of standing (s)	24/24	31 ± 6^a^	24/24	15 ± 4^b^
Latency to first walking (s)	12/24	6 ± 9	14/24	9 ± 5
Duration of walking (s)	12/24	5 ± 3	14/24	2 ± 1
Latency to first sitting (s)	12/24	31 ± 3^a^	12/24	14 ± 5^b^
Duration of sitting (s)	12/24	3 ± 2	12/24	3 ± 3
Latency to first lying (s)	24/24	34 ± 5^a^	24/24	17 ± 3^b^
Duration of lying (s)	24/24	310 ± 5^a^	24/24	328 ± 3^b^
**Events**				
Sniffing (s)	24/24	18 ± 3^a^	24/24	7 ± 2^b^
Latency to first retreat attempt (s)	22/24	22 ± 6^a^	20/24	10 ± 4^b^
Number of retreat attempts	22/24	2 ± 1	20/24	2 ± 1
Latency to first gasp (s)	24/24	23 ± 4^a^	24/24	9 ± 3^b^
Number of gasps	24/24	30 ± 9^a^	24/24	14 ± 3^b^
Latency to first lateral head movement (s)	8/24	24 ± 10	12/24	14 ± 6
Number of lateral head movements	8/24	2 ± 1	12/24	1 ± 1
Latency to first jump (s)	12/24	34 ± 5^a^	11/24	14 ± 2^b^
Number of jumps	12/24	1 ± 1	11/24	2 ± 2
Latency to first muscular contraction (s)	21/24	36 ± 4^a^	24/24	20 ± 6^b^
Number of muscular contractions	21/24	4 ± 2	24/24	3 ± 2
Loss of posture (s)	24/24	44 ± 5^a^	24/24	26 ± 5^b^
Number of escape attempts	0/24	–	0/24	–

*^1^First behavior when entering the gondola was always standing*.

*^2^Number of pigs showing the specific behavior*.

*T = 0 is start descending of the gondola into the well*.

*^a,b^Means with different letters are significantly different (*P* < 0.05)*.

The time between first and last occurrence of gasping and muscular contractions was also assessed. The time between first and last gasp was longer (*P* < 0.001) in 80C pigs (206 ± 77 s) compared to 95C pigs (111 ± 26 s). The number of gasps while conscious, however, did not differ between 80C pigs (6 ± 3) and 95C pigs (5 ± 3). The time between first and last muscular contraction did not differ between 80C pigs (47 ± 40 s) and 95C pigs (59 ± 55 s).

Immediately after the end of the exposure, none of the 95C pigs showed a corneal reflex or breathing. In two 80C pigs, in two different runs, gasping was observed post stunning and these pigs were immediately immersed in CO_2_ for another 5 min. No corneal reflex was observed in these pigs. Concentrations of CO_2_ during these two runs were 79.2 and 82.4% CO_2_.

#### Behavior in Relation to the EEG

**Figure 3:**
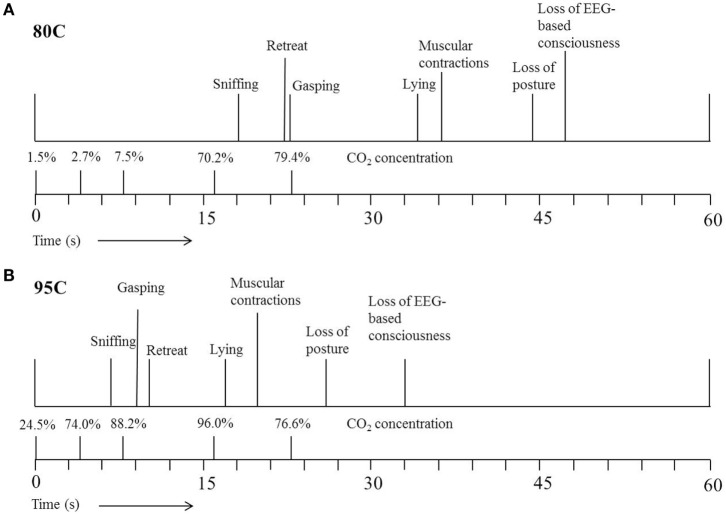
**Average latency (s) to the different behaviors expressed by pigs exposed to 80% CO_2_ [80C (A)] or 95% CO_2_ [95C (B)]**. *T* = 0 indicates start descending of the gondola into the well pre-filled with CO_2._ The actual CO_2_ concentration was measured once at five different time points during descending of the gondola into the well.

Figure 3 shows the average latencies to the different behaviors and EEG-based loss of consciousness expressed by 80C (Figure 3A) or 95C (Figure 3B) pigs. Both 80C and 95C pigs showed a similar sequence of behaviors when exposed to the CO_2_.

**Figure 4:**
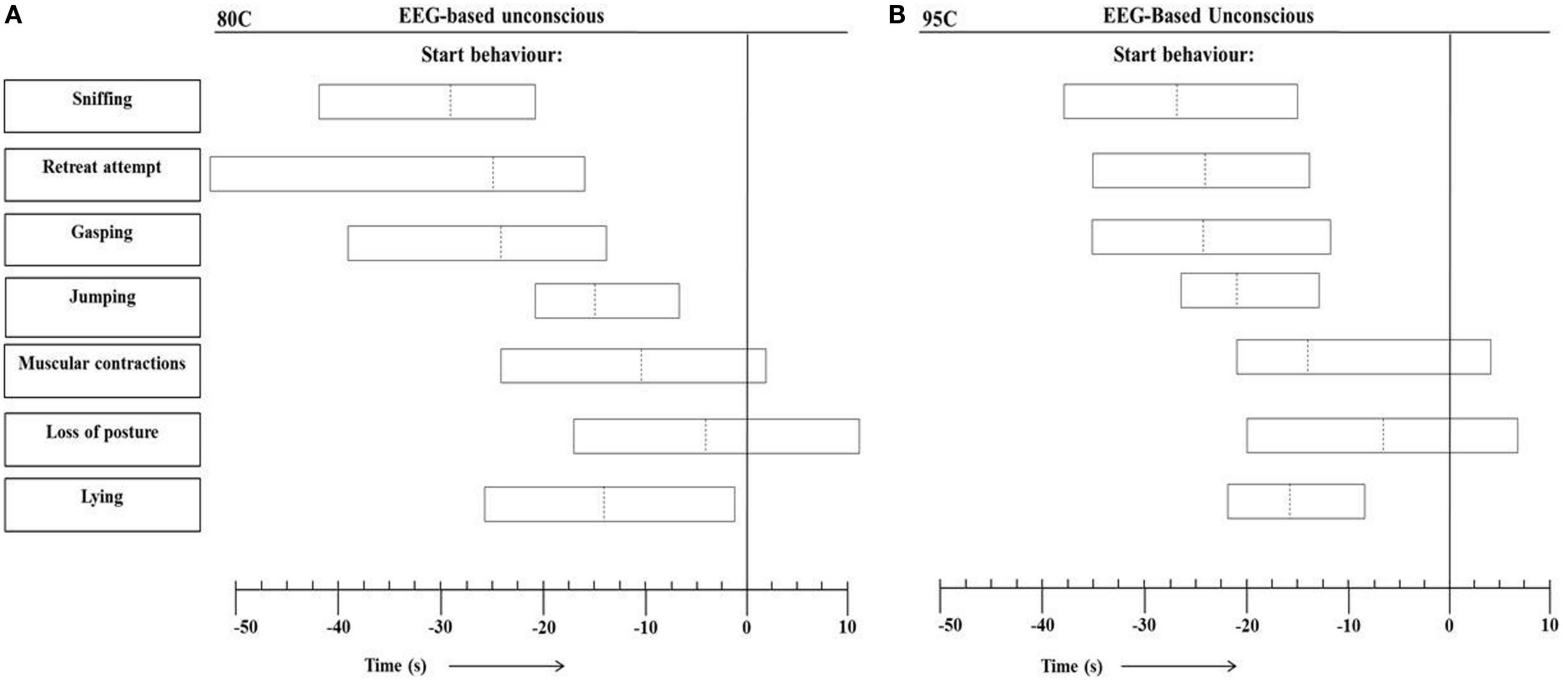
**Range of individual time points at which the different behaviors occurred in relation to EEG-based loss of unconscious (*T* = 0) during CO_2_ stunning in pigs exposed to 80% CO_2_ [80C, *n* = 24 (A)] or 95% CO_2_ [95C, *n* = 24 (B)]**. Dotted lines indicate average values of all observations and left and right vertical lines of each block indicate minimum and maximum values for all observations.

Figure 4 presents the range of individual time points at which the different behaviors started in relation to onset of EEG-based unconsciousness, based on visual assessment of EEG recordings, in 80C pigs (Figure 4A) and 95C pigs (Figure 4B). In both 80C and 95C pigs, sniffing, latency to first retreat attempt, gasping, jumping, and lying occurred before EEG-based loss of consciousness was observed. Muscular contractions were observed in 88 and 95% of 80C and 95C pigs, respectively, before EEG-based loss of consciousness. Loss of posture was observed in 63 and 81% of 80C and 95C pigs, respectively, before EEG-based loss of consciousness. Latencies to behaviors relative to onset of unconsciousness did not differ between both treatments.

## Discussion

According to EEG data and behavioral observations, consciousness was reported to be lost during CO_2_ stunning (80–90% by volume in air) on average 30 s following onset of exposure based on both EEG data and behavior (7, 10).

The time to loss of consciousness during CO_2_ stunning, however, remains debated (30) and may depend on multiple factors, including the CO_2_ concentration applied and experimental design. An abrupt exposure to the gas mixture is known to induce a more rapid loss of consciousness in comparison with gradual immersion (31). Some of the previous studies simulated commercial conditions [e.g., Ref. (9, 32)] where pigs were immersed gradually to the required concentration at the bottom of a well. Part of the descending time was, therefore, taken up with transit through the interface between air and CO_2_, which is expected to prolong the conscious period. Based on EEG recordings, the pigs in the current study immersed in 80C and 95C lost consciousness, after on average 47 and 33 s, respectively, after descending the gondola into the well. Our results are similar to those reported by Llonch et al. (33) who reported loss of consciousness, based on EEG activity, 37.6 s after starting descending the gondola in a well pre-filled with 90% CO_2._ Descending the gondola into the well also took 23 s in that experiment. During experiments performed by Forslid (24) and Raj (34), animals were immersed immediately (24) or within 10 s (34) in a box that contained the modified atmosphere. Forslid (24) concluded that low-frequency activity dominated the EEG trace, indicative of unconsciousness, after 23–28 s in pigs immersed in 80C. Raj (34) observed loss of posture 17 ± 3 s after pigs were exposed to the highest (80–90%) CO_2_ concentration. Rodriguez et al. (9), on the other hand, monitored brain activity using auditory evoked potentials and concluded time to loss of consciousness to be 60 s after being submersed into a well pre-filled with 90% CO_2_. It should be noted, however, that the use of absence of evoked responses may provide more conservative times to loss of consciousness compared to loss of spontaneous EEG activity (35, 36). Another possible explanation for the variety in time to loss of consciousness is that pigs differ in their responses to CO_2_ exposure and that these responses could be breed dependent, but also depend on the way in which animals are handled pre-slaughter (3, 37, 38).

During our study, time taken to loss of consciousness decreased with as CO_2_ concentration increased as previously reported by Raj and Gregory (7) and EFSA (10). Pigs subjected to 95C in the current study, were exposed to 74% CO_2_ at 4 s after starting descending of the gondola, whereas pigs subjected to 80C were exposed to only 3% CO_2_ at a similar depth. A faster and deeper respiration pattern observed in high CO_2_ concentrations results in an increased intake of CO_2_ and thereby increased efficiency of the stunning method, whereby the induction period is reduced and consequently time to loss of consciousness (4).

Pigs exposed to 80C and 95C revealed an iso-electric EEG after 75 and 64 s, respectively, indicating severely reduced central nervous system activity. Exposure to high CO_2_ concentrations is still reversible after 1–2 min (6, 24), but prolonged exposure to 80C resulted in death after approximately 2–3 min in the majority of the pigs in a study by Raj (34). In the current study, 95C pigs had all died after the long exposure to CO_2_, confirmed by the absence of brain activity, breathing, and brain stem reflexes. Two 80C pigs, however, displayed signs of gasping, although both animals showed an iso-electric EEG. Because recovery from CO_2_ stunning was not part of this study, these pigs were immediately immersed for another 5 min in 80C. Since no corneal reflex after ascending of the gondola was observed in these pigs, it is likely that these gasps were only rudimentary brain stem activity and not signs of recovery from the CO_2_ exposure.

Brain activity, as presented in an EEG, is considered the most objective method available for the assessment of unconsciousness. This method, however, is only used for research objectives because its application holds numerous challenges during stunning and slaughter of livestock. One of the challenging aspects is that the EEG can be influenced by artifacts that are animal- or technical related (39). Experimental controlled situations provide a better environment to limit these artifact sources than slaughter plants. Several studies on stunning and slaughter report disconnected electrodes or disrupted EEG activity in 9–71% of the animals (40–42). The CO_2_ stunning procedure itself provides an additional challenge, as animals can move freely and (extensive) muscular contractions can easily disturb the EEG signal. Visual appraisal of EEG activity to assess the state of (un) consciousness has been applied during studies in poultry (43), sheep (27), and veal calves (28). In addition to visual appraisal, EEG signals can be assessed using fast Fourier transformation (FFT). The output thereof represents the frequency composition of the signal, or alternatively formulated, how much power is presented in the different frequency bands. As this output is automatically derived, its results are considered more objective than visual appraisal. FFT analyses, however, necessitate a clean and stable EEG signal. Removal of artifacts is possible using certain types of filters, but this can also remove important information from the EEG trace as movement artifacts often occur in the 0–4 Hz range (Gerritzen, personal communication). Previous work by our group assessed the relation between onset of the different EEG stages, based on visual assessment of EEG activity, and spectral variables “Total Power” and “Spectral Edge Frequency,” during propofol anesthesia in sheep (27). There were strong correlations between onset of the different EEG stages based on visual EEG assessment and these two spectral variables ranging from 0.68 to 0.95 (Verhoeven, unpublished results). This supports the validity of visual assessment of EEG traces as conducted in the present study. It was not possible to perform continuous FFT analyses due to the muscular contractions of pigs that influenced the EEG traces.

Descending and ascending of the lift has been thought to induce fear in pigs. Holst (2002, cited by EFSA, 2004) found that 77% of the pigs stood motionless (freezing) in the gondola when lowered in atmospheric air (10). The majority of these pigs started exploring the gondola while it was stationary. Based on these findings, EFSA (10) concluded that the vertical movement of the gondola itself induces fear in the pigs (10). Dalmau et al. (44), on the other hand, found that the time taken to cross the raceway and enter the gondola did not differ between subsequent training sessions and Velarde et al. (3) found an increased percentage of pigs voluntarily entering the gondola in subsequent training sessions (3, 44). Subjective observations during air treatment days in the current study indicated no differences in behavior of pigs entering the gondola on these days. The majority of pigs, however, stood motionless, in the gondola while descending and ascending. It cannot be excluded, though, that the animals stood still to keep balance while moving up and down and not because animals were fearful (Bolhuis, personal communication). Though it is difficult to perceive what pigs experience during the CO_2_ induction period, the general opinion is that pigs respond aversively when exposed to high concentrations of CO_2_. Velarde et al. (3) found increased times taken to cross the raceway and enter the gondola when pigs were exposed to a CO_2_ treatment compared to an air treatment and when exposed repeatedly to 70 or 90% CO_2_. Therefore, exposure to CO_2_ was considered more aversive than exposure to atmospheric air. During the current study, there was a clear difference in pig behavior when exposed to atmospheric air or CO_2_. None of the pigs were observed sitting or lying and no gasping, jumping, muscular contractions, or escape attempts were observed on these 2 days. Exposure to CO_2_ stimulates respiration and pigs start to hyperventilate (7). In humans, this is described as breathlessness that is known to increase with blood carbon dioxide levels (45). Moreover, CO_2_ is an acidic gas with a high solubility that together with water forms carbonic acid. With CO_2_ stunning, carbonic acid is formed when the CO_2_ dissolves in water from mucous membranes. It is, therefore, believed that CO_2_ causes irritation and pain in the lining of the nasal cavity when inhaled (46, 47).

There is continuing debate on which CO_2_ concentrations are most aversive to pigs since their behavioral responses vary with different CO_2_ concentrations. Signs of aversive behaviors include lateral head movements, retreat, and escape attempts (7, 9, 12, 18). In a study by Rodriguez et al. (9), lateral head movements were the first behavior of pigs, on average, 10 s after initial exposure to 90% CO_2_. Hartung et al. (26) stated that the head movements were a clear indication that the animal had detected the gas and responded aversively to it. When confronted with an unpleasant situation, the response of a pig is often to back away (retreat) or escape (18). Dodman (18) observed that all pigs showed retreat attempts in 50–55% CO_2_ and 37% of the pigs showed this response in 76–80% CO_2_. In the current study, 92 and 83% of the pigs exposed to 80C and 95C, respectively, showed at least one retreat attempt. Although the analgesic effect of CO_2_ has been demonstrated for higher concentrations (10), the initial acute exposure to high carbon dioxide levels may induce an aversive response. This latter transient effect has been attributed to the irritating, and potential painful, influence on the mucous membranes. In the current study, no differences in the percentage of pigs showing retreat attempts could be determined between pigs exposed to 80C and 95C_._ In two studies by Raj and Gregory, none of the pigs showed escape attempts when exposed to CO_2_ concentrations lower than 30% or higher than 80% (7, 12). The majority of pigs in a study by Velarde (3), however, attempted to escape when exposed to 90% CO_2_. During the current study, no escape attempts were observed. It is possible, however, that escape attempts were difficult to observe and this behavior is in the current study marked as jumping or muscular contractions.

Gasping has not been considered an aversive behavior as it occurs due to residual medullary activity in the brainstem when it becomes hypercapnic (48). It is a physiological reaction associated with breathlessness during the inhalation of high concentrations of CO_2_. All of the 80C and 95C pigs showed gasping before loss of consciousness, but the latency to gasping was shorter in 95C pigs than in 80C pigs. Duration from latency to gasping and loss of consciousness and the number of gasps while conscious, however, were similar in both groups. Figure 3 shows that gasping occurred closely in time with retreat attempts. It may be assumed that gasping does compromise animal welfare in conscious pigs, because it is associated with a sense of breathlessness (16).

Muscular contractions are observed in the majority of pigs exposed to high CO_2_ concentrations and it has been heavily debated whether they occur before or after animals have lost consciousness. It has been suggested that muscular excitations are the result by the lack of modulation of the caudal reticular formation from higher centers, particularly the cerebral cortex and physical activity during CO_2_ exposure might be an aversive response to the rostral reticular formation (10). Zeller et al. (49) and Rodriguez et al. (9) stated that the respiratory distress was induced by inhalation of gas. Dalmau et al. (44) observed that time taken to cross the raceway and enter the gondola was lower in pigs without muscular excitations in the previous sessions than pigs with a high intensity of these muscle excitations, supporting the hypothesis that muscular excitations induce traumatism and pain. In that same study, one pig was replaced in the first trial due to lameness. From these results, it was concluded that pigs might have associated the pain after or during the muscular excitation phase with the stunning system and consequently refused to enter the gondola in the following session. In this study, muscular contractions were observed in the majority of the pigs before they were considered unconscious and possibly compromising animal welfare. Since pigs were only exposed to the CO_2_ once, it was not possible to observe their response to the stunning system a second time.

Loss of posture has been suggested as first indicator of onset of unconsciousness (7). Raj and Gregory (7) defined it as the time to loss of posture (a recumbent state), whereas in the present study it was defined as “the pig is in a recumbent position with no sign of control of posture.” The latter would indicate that a pig in the current study that would lay on the floor, but lifts it head up or still look up or around would not be considered having loss of posture and would be considered lying. Loss of posture was on average observed 10 s before loss of consciousness. When taking lying as a first indicator of onset of unconsciousness, it was on average scored 15 s before loss of consciousness. Lying, however, was easier to score than loss of posture. None of the behaviors scored were able to exactly pinpoint time to loss of consciousness, but the loss of posture was on average closest and considered the first indicator of onset of unconsciousness.

The most important issue for welfare is what an animal experiences while conscious. The present study indicates that pigs respond aversively by means of lateral head movements, retreat attempts, and possible jumping when exposed to 80C or 95C. Muscular contractions were observed in conscious pigs exposed to either 80C or 95C and this may compromise animal welfare. The number of behaviors and time from first occurrence of a behavior relative to loss of consciousness, however, did not differ between the pigs exposed to 80C or 95C in the present study. The findings provide little reason to conclude on a behavioral basis that these atmospheres are greatly different in their impact on pig welfare.

## Author Contributions

MV was involved in all steps leading to this manuscript and was responsible for the practical part of the study. AV contributed to the preparation of the practical part of the study. MG, AV, LH, and BK contributed significantly to the discussion of the subject, and the development, writing, and final version of this paper.

## Conflict of Interest Statement

The authors declare that the research was conducted in the absence of any commercial or financial relationships that could be construed as a potential conflict of interest.
